# The impact of specific cytokine directed treatment on severe COVID-19

**DOI:** 10.1038/s41375-021-01411-1

**Published:** 2021-09-07

**Authors:** P. A. Reuken, M. M. Rüthrich, A. Hochhaus, J. Hammersen, M. Bauer, P. La Rosée, F. La Rosée, A. Stallmach

**Affiliations:** 1 Klinik für Innere Medizin IV, Universitätsklinikum Jena, Friedrich-Schiller-Universität Jena, Jena, Germany; 2 Klinik für Innere Medizin II, Universitätsklinikum Jena, Friedrich-Schiller-Universität Jena, Jena, Germany; 3 Klinik für Anästhesiologie und Intensivmedizin, Universitätsklinikum Jena, Friedrich-Schiller-Universität Jena, Jena, Germany; 4 Klinik für Innere Medizin II, Schwarzwald-Baar Klinikum, Villingen-Schwenningen, Germany; 5 Medizinische Fakultät Carl Gustav Carus, Technische Universität Dresden, Dresden, Germany

**Keywords:** Infectious diseases, Risk factors

## To the Editor:

Severe acute respiratory syndrome coronavirus 2 (SARS-CoV-2) in select patients elicits a cytokine storm, which accounts for disease progression and the need for intensive care therapy. This phase of the corona virus disease (COVID-19) is characterized by hyperinflammation driven by an overwhelming host immune response [1]. It is treated with dexamethasone as standard of care [2], however, some patients progress despite this therapy. Therefore, we read with great interest the study by Neubauer et al. [3] reporting beneficial effects of the JAK-inhibitor ruxolitinib in severe COVID-19, which supports previous results of ruxolitinib in COVID-induced hyperinflammation [4]. In parallel, based on beneficial experiences in patients with inflammatory bowel disease and COVID-19, Stallmach et al. demonstrated a reduction in mortality in seven COVID-19 patients treated with infliximab, an anti-TNF-antibody [5].

Currently, the choice of an anti-inflammatory agent is based on the clinical decision and experience of the treating physician, as direct head-to-head comparisons are absent. Therefore, we aimed to evaluate the outcome of patients treated with ruxolitinib, infliximab, or without anti-inflammatory therapy that exceeded the standard of care, i.e., dexamethasone in a retrospective matched pair design based on recently published cohorts [4, 5] and additional patients treated since the publication of these cohorts in a matched pair design.

Patients hospitalized for severe, PCR-proven COVID-19 with and without anti-inflammatory treatment were retrospectively analyzed at Jena University Hospital (controls and infliximab patients) and Schwarzwald-Baar-Klinikum (ruxolitinib patients). Patients were matched to anti-inflammatory treated patients 1:1 with respect to age, sex, and WHO score. Administration of infliximab or ruxolitinib was based on the decision of the treating physician. In patients receiving anti-inflammatory therapy, day 1 was defined as the start of therapy, and as the day of admission in control patients. The study was approved by the local ethics committee.

A total of 77 patients were included in our analysis. Nineteen received infliximab (5 mg/kg body weight as a single dose), 20 were treated with ruxolitinib (7.5 mg bid with individual dose adaption according to efficacy, median duration 10.5 days, range 5–20 days), and 38 patients received no specific anti-inflammatory treatment other than corticosteroids. In all three groups, the majority of patients were male, while the median age was 59 years in the infliximab group and 66 years in the ruxolitinib and control groups (*p* = 0.536). The majority of patients were concomitantly treated with corticosteroids: 72.2% in the infliximab group and 69.4% in the control group received dexamethasone, and 70% in the ruxolitinib group were treated with prednisolone. Remdesivir was used in infliximab patients (66.7%) and control patients (63.9%) only (*p* = 0.544). In the ruxolitinib group, no patient received remdesivir, as all of them were included in April and May 2020, before the approval of remdesivir. Details on the baseline characteristics are presented in Table 1.

**Table 1:** Baseline characteristics of the patients.

	IFX (*n* = 19)	Controls (*n* = 38)	RUXO (*n* = 20)	*P* value
Age (years)	59 (53; 72)	66 (54; 72)	66 (59; 70)	0.536
Sex male, *n* (%)	15 (78.9)	30 (78.9)	13 (65.0)	0.384
Ventilation, *n* (%) including:	12 (66.6)	29 (79.2)	16 (80.0)	0.469
NIV, *n* (%)	6 (33.3)	9 (23.7)	12 (60.0)	
Mechanical ventilation, *n* (%)	6 (33.3)	20 (55.5)	4 (20.0)	
Corticosteroids, *n* (%)	13 (72.2)	25 (69.4)	14 (70.0)^b^	0.948
Remdesivir, *n* (%)	12 (66.7)	25 (63.9)	0	0.544^a^
IL6 day 1 (ULN)	11.5 (8.5; 22.7)	11.3 (5.6; 25.9)	7.1 (4.3; 13.5)	0.429
IL6 day 7 (ULN)	4.7 (1.8; 9.9)	4.1 (1.4; 12.1)	1.0 (0.3; 4.9)	0.945
CRP day 1 (mg/l)	165.6 (111.5; 267.1)	151.7 (101.6; 217.7)	122.3 (70.7; 195.9)	0.209
CRP day 7 (mg/l)	90.3 (61.9; 169.4)	120.8 (56.8; 162.0)	50.0 (24.1; 103.4)	0.025
WBC day 1 (/nl)	8.9 (5.9; 13.5)	7.5 (5.0; 12.4)	7.9 (6.0; 11.8)	0.692
WBC day 7 (/nl)	7.0 (5.5; 10.0)	7.2 (5.1; 11.9)	8.1 (7.2; 12.2)	0.199
Lymphocytes day 1 (/nl)	0.51 (0.39; 0.87)	0.64 (0.41; 0.91)	0.95 (0.66; 1.23)	0.011
Lymphocytes day 7 (/nl)	0.83 (0.40; 1.32)	0.62 (0.43; 1.13)	1.40 (1.01; 2.09)	0.001
Ferritin day 1 (µg/l)	2538 (1563; 2948)	1695 (908; 2347)	1501 (1186; 2367)	0.198
Ferritin day 7 (µg/l)	2294 (1269; 3758)	1458 (933; 2589)	1783 (1361; 2336)	0.256
D-Dimer day 1 (µg/l)	543.0 (373.8; 745.8)	437.0 (303.0; 2095.0)	1670 (1167.5; 2487.5)	0.001
D-Dimer day 7 (µg/l)	557.5 (330.8; 3750.1)	460.0 (250.0; 1227.0)	1095 (905; 3245)	0.006
Creatinine day 1 (µmol/l)	70.7 (56.0; 102.3)	88.0 (66.5; 147.5)	89.9 (73.1; 124.2)	0.096
Creatinine day 7 (µmol/l)	66.5 (55.3; 82.5)	88.0 (58.0; 128.0)	85.9 (75.3; 110.4)	0.048
CIS day 1	11 (10; 12)	10 (9; 11)	12 (11; 13)	0.001
CIS day 7	9 (7; 12)	9 (7; 13)	8 (5; 10)	0.060
30-day-mortality	1/19 (5.2%)	11/38 (28.9%)	3/20 (15%)	0.080

All data are presented as mean and 1st/3rd quartile or as absolute number and percentage.

*NIV* non-invasive ventilation, *CRP* C-reactive protein, *IL6* Interleukin 6, *ULN* upper limit of normal, *WBC* white blood cells, *CIS* COVID Inflammation Score.

^a^Only IFX vs. no IS was used for calculation of the *p* value, as no RUXO patient received remdesivir.

^b^One patient received tocilizumab 600 mg once.

**Fig. 1:**
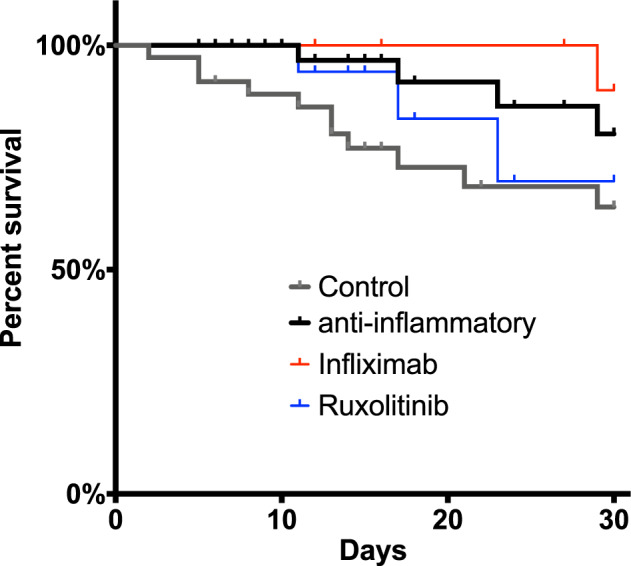
Kaplan–Meier analysis of 30-day mortality in patients treated with anti-inflammatory biologicals, JAK inhibitors or without anti-inflammatory therapy. Data were censored at hospital discharge. *p < 0.05 in log-rank-test.

Within 30 days after inclusion, 15 of the patients died (19.4%), including 4 patients with intensive anti-inflammatory therapy (10.3%) and 11 patients in the control group (28.9%) (*p* = 0.041 in log-rank test). Of the 4 patients who died after intensive anti-inflammatory treatment, 1 patient received infliximab (5.2%), and 3 patients received ruxolitinib (15.0%). (Fig. 1) Notably, mortality in the anti-inflammatory-treated patients was lower despite a higher degree of hyperinflammation, as indicated by the recently introduced covid inflammation score (CIS; 12 vs. 10 points, *p* < 0.001 on day 1). Comparing the different anti-inflammatory regimens, we did not observe a significant difference in 30-day mortality between patients treated with ruxolitinib or infliximab (Supplementary Fig. 1, *p* = 0.607).

At day 5, the CIS decreased in all groups, and the highest decrease was found in the ruxolitinib group (−4 points to a median of 8 points), which was interestingly the group with the highest pretreatment CIS. The decrease in CIS was significant in ruxolitinib-treated patients (*p* < 0.001) and showed a trend in the infliximab group (*p* = 0.082), while there was no effect in the control group (*p* = 0.992). (Table 1**)**.

Our data are consistent with the notion that the addition of a specific anti-inflammatory therapy to corticosteroids in patients with severe COVID-19-induced hyperinflammation is associated with lower mortality than that in age- and sex-matched controls without intensive anti-inflammatory therapy. While the use of corticosteroids, specifically dexamethasone, entered treatment guidelines after the publication of the Recovery data in 07/2020 [2], there is an ongoing debate regarding drugs that can be used in patients needing intensified and specific anti-inflammatory therapy if hyperinflammation persists or increases after starting steroids. In our study, we demonstrated that both JAK inhibitors and anti-TNF antibodies can reduce the risk of death within 30 days in patients with severe COVID-19, which is also supported by a recent meta-analysis of ruxolitinib in COVID-19 [6].

Contrasting that, a recent randomized controlled trial did not find an impact of ruxolitinib on mortality but only on the time to recovery [7]. One important aspect in anti-inflammatory therapy for COVID-19 is timing of the drug in relation to hyperinflammation. In the Recovery trial, a too early anti-inflammatory treatment with dexamethasone in patients without need for oxygen supply was associated with an increased mortality [2]. In contrast, a treatment delay can minimize the therapeutic effect of anti-inflammatory drugs due to already established organ failure. In our study, hyperinflammation was highest in ruxolitinib patients, as indicated by the CIS [4]; therefore, one would have expected a higher mortality compared to the controls.

A main difference despite the target molecule between JAK inhibitors and TNF antibodies is the route of administration. The anti-TNF antibody infliximab was given as a single dose via intravenous infusion; in contrast, the JAK-inhibitor ruxolitinib was administered orally twice a day for up to 20 days and tapered according to the interdisciplinary COVID board recommendation [8]. The single-shot administration of an anti-inflammatory agent may theoretically have a benefit in COVID-19 patients as (i) the full dose is given at the beginning of therapy with a possibly more rapid effect and (ii) treatment may be beneficial in critically ill patients, where timing is crucial and intestinal malfunction may impact proper absorption. Mortality, however, was lower in the ruxolitinib-treated group, and the decline in hyperinflammation was most pronounced in this group, indicating no major impact of oral vs. i.v. application of specific anti-inflammatory therapy.

Other drugs investigated in the context of COVID-19 include IL6 blockade with tocilizumab, but again, the results are inconclusive. While Zhao et al. reported a benefit when treating patients with tocilizumab [9], Burlacu et al. did not find a benefit from tocilizumab treatment in severe COVID-19 [10]. However, most of these reports compare one anti-inflammatory agent against dexamethasone or the standard of care. A direct head-to-head comparison between two or more cytokine-directed agents is still missing.

Our study has several limitations. First and most importantly, due to the retrospective design, we cannot exclude selection bias, as the choice of drug was made by the treating physician based on availability, local standards, and personal experience. Second, there may be factors other than anti-inflammatory treatment that may have influenced the outcome of the patients.

Nevertheless, our study supports the concept of specific anti-inflammatory treatment in patients with COVID-induced hyperinflammation despite dexamethasone treatment. Randomized controlled trials investigating ruxolitinib and infliximab are urgently needed and are already recruiting patients. However, until these results are available, treatment with either infliximab or ruxolitinib according to the personnel experience of the treating physician can be justified in some COVID patients, especially when they present with hyperinflammation.

## Supplementary information

Supplemental Figure

## Data Availability

Data underlying this manuscript are available from the corresponding authors upon reasonable request.
