# Supplementary figures and images for: The impact of specific cytokine directed treatment on severe COVID-19
Source: Leukemia. 2021 Sep 7;35(12):3613–5. doi: 10.1038/s41375-021-01411-1 (PMC8422045; doi:10.1038/s41375-021-01411-1)

Survival percentage

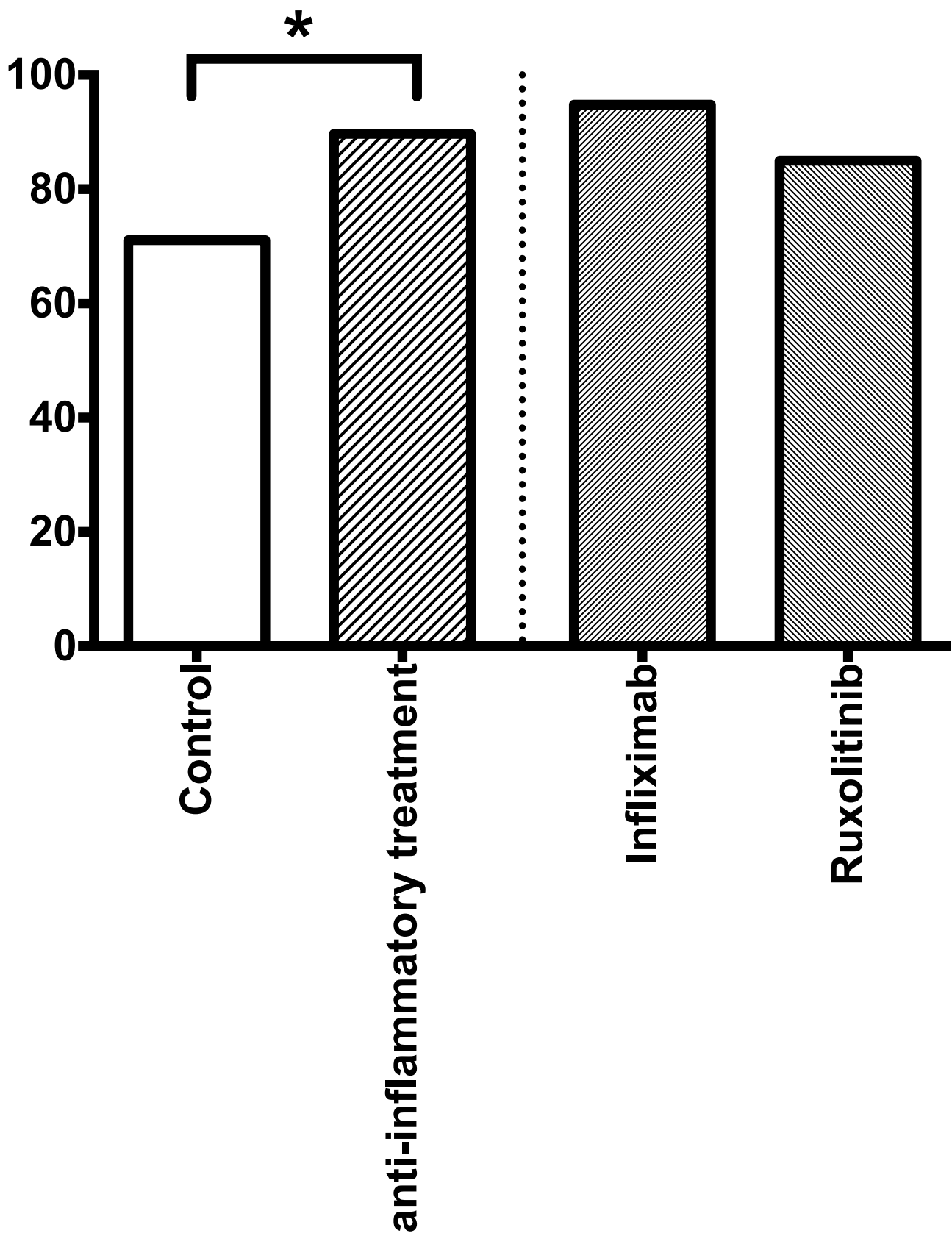

Supplement: Supplementary file 1 — Supplemental Figure [file 41375_2021_1411_MOESM1_ESM.pdf]
